# Who is bearing the financial burden of non-communicable diseases in Mongolia?

**DOI:** 10.7189/jogh.08.010415

**Published:** 2018-06

**Authors:** Otgontuya Dugee, Enkhtuya Palam, Bayarsaikhan Dorjsuren, Ajay Mahal

**Affiliations:** 1 School of Public Health and Preventive Medicine, Monash University, Melbourne, Australia; 2 Public Health Institute of the Ministry of Health of Mongolia, Ulaanbaatar, Mongolia; 3 Department of Health Systems Governance and Financing, World Health Organization, Geneva, Switzerland; 4 Nossal Institute for Global Health, University of Melbourne, Melbourne, Australia

## Abstract

**Background:**

Non-communicable diseases (NCDs) pose a formidable health and development challenge for low- and middle-income countries (LMICs). However, translating this challenge into resource allocation is seriously constrained by a lack of country specific evidence on NCD financing and its distributional implications. This study estimated expenditures associated with NCDs in Mongolia and their distributions across socioeconomic groups, focusing especially on private out-of-pocket (OOP) spending on the major NCDs.

**Methods:**

Secondary data analysis of multiple data sources on NCD related health service use and expenditures including detailed administrative data, World Health Organization STEPwise approach to Surveillance (STEPs) survey for Mongolia, and household surveys. Sample-weighted estimates of OOP expenditures for NCDs were constructed using STEPs data. OOP payments per discharge and per outpatient visit were estimated by condition and type of service provider, and survey data on utilization, after adjusting for utilization in administrative records.

**Results:**

NCDs in Mongolia accounted for more than one-third of total health expenditures in 2013. A significant fraction of this expenditure was borne by households in the form of OOP spending. CVD-related health spending is the major driver of NCD-spending in Mongolia, accounting for about 24.2% of total health expenditure. OOP health payments, largely driven by outpatient diagnostics and drugs, were incurred disproportionately by the better-off, seeking more specialist services and better quality private care.

**Conclusion:**

A high share of OOP spending for NCDs in Mongolia, which ostensibly enjoys universal health coverage, provides a cautionary tale for LMICs in a similar situation. Improvement in the quality of services at the primary care level and rural health care facilities, where the poor mainly attend, is desirable together with an effective exemption policy for user fees at higher level hospitals.

The share of non-communicable diseases (NCDs) in the disease burden in many low-and middle-income countries (LMICs) is approaching that of high-income countries [1], mostly due to cardiovascular diseases (CVD), cancer, chronic respiratory diseases, and diabetes [2]. Universal Health Coverage (UHC) is considered a key element of sustainable human development goals [3] and, given the growing significance of NCDs, it is increasingly recognised that prioritizing NCDs may create opportunities to more effectively attaining UHC in LMICs [4-6].

NCD-related objectives are also embedded in the Sustainable Development Goals in recognition of its adverse effects on development [2]. Although NCDs pose a formidable health and development challenge [7,8] allocation of additional resources for action against NCDs in LMICs is constrained by a lack of country-specific evidence, domestic resource constraints and communicable and maternal and child health conditions that also warrant policy attention [9-12]. Information on the distributional implications of NCDs is also limited, particularly for economic outcomes.

We are aware of only three Asian countries (India, Mongolia and Sri Lanka), that have published information on NCD financing [13-16]. In Sri Lanka and India, a lack of reliable administrative records of sufficient detail has meant relying on data not geared towards gathering NCD-specific information. Furthermore, within-country inequalities in the use of NCD-related health care services [17] and distribution of NCD expenditures across socioeconomic groups are also unexplored. One might suspect, for example, that the economic impact of NCDs on poorer individuals is likely to be more severe than their richer counterparts [17] because of lower treatment coverage [18], less effective management of complications [19] and shorter survival [20]. Recently, Dugee et al. [16] estimated total public spending on NCDs in Mongolia, using a national health accounts methodology, but their study lacks a major component of NCD spending, namely private out of pocket (OOP) payments.

We estimate expenditures associated with NCDs in Mongolia and their distribution across socioeconomic groups, with a specific focus on private spending on NCDs. Mongolia is a particularly interesting case to study, given it is a middle income country similar to Malaysia and Sri Lanka [21] with a relatively high share of public spending on health, while simultaneously facing budgetary constraints to achieve universal health coverage. And as in Malaysia, a growing private health care sector in Mongolia has emerged to meet the demands of patients needing greater personal attention and shorter waiting times. Crucially, Mongolia routinely collects high quality administrative data on health service use, along with periodic surveillance data (WHO STEPs surveys) specifically geared towards gathering information on service use and expenditures on NCDs.

Mongolia inherited a Semashko-type health system reflecting its close ties with the Soviet Union. By this we mean a multi-tiered system that was completely state-controlled and owned, including all levels of hospitals and practicing doctors. Health care was free for all [22,23]. Mongolia transitioned to a more market-based health care delivery and financing system in the 1990s, with some separation of financing from provision with the introduction of social insurance, and the emergence of private sector providers [24,25].

More recently, the Mongolian Health Act of 2006 introduced a core package of essential services to be directly funded by the state budget, and a complementary package of services financed by a mix of social health insurance (SHI), out-of-pocket (OOP) payments and donor funds [26]. The essential services package has multiple components, including primary care with GPs (general practitioners) in the city and province centres, and preventive and curative services in “*soum*” health centres (health centres in rural areas with some inpatient beds). The complementary package covers secondary and tertiary care services, funded mainly from SHI and general revenues, but supplemented by co-payments and user charges. In Mongolia, OOP payments comprise official co-payments and user fees in public facilities, and direct payments to private facilities, with some informal fees [24]. Public sector hospitals providing secondary and tertiary care collect household payments for inpatient services and specialist outpatient care, including diagnostics and tests. Co-payments approved under Mongolian Health Insurance Law amount to about 10-15% of the SHI reimbursements for public sector inpatient services, with exemptions for children under 18, retirees, disabled and some others. The revised Health Act 2006 also allowed hospitals to charge for some elective services [24,27]. OOP payments in public hospitals are recorded as revenue to facilities and reported to the treasury as revenue from “main activities,” amounting to about 3.0% of public expenditures on health in 2013, although this figure may be an underestimate [24].

Details of public funding on NCDs have been reported previously [16]. Cancer inpatient care in public facilities is fully subsidized by the state budget, whereas patients with cardiovascular disease (CVD), diabetes mellitus, chronic obstructive pulmonary disease (COPD) and asthma incur co-payments for inpatient care. Outpatient drugs to treat the end-stages of cancer and diabetes mellitus are funded from the state budget, but face budgetary constraints [24,27]. There are co-payments, however, for outpatient prescription drugs (in the essential drug list) prescribed by primary care physicians, [28,29]. In addition, there are private clinics for which OOP is incurred, and private hospitals that are paid for by a mix of OOP and social insurance payments. Accredited private hospitals are also reimbursed by SHI, but at a rate one-half that of public hospitals [28,29].

In 2013, Mongolia spent 4.2% of its gross domestic product (GDP), or about US$ 185 per capita on health care. Public expenditures, including expenditures financed from SHI, accounted for 52.5% of total health expenditure (THE), mostly for allocations to public facilities. Private expenditures on health services were 44.3% THE in 2013 (mostly household OOP payments) in 2013 [30]. Population health indicators have improved over time, with life expectancy at birth rising from 60.3 years in 1990 to 69.4 years in 2014, but NCDs have emerged as a major health challenge. NCDs accounted for 61.5% of all cause deaths in Mongolia 2013, with CVD being the leading cause of death, accounting for 37.4% of all deaths [31]. The latest World Health Organization report on NCDs estimates that the probability of dying between ages 30–70 from major NCDs in Mongolia is 32% compared to the global average of 19% [1].

## METHODS

### Data

Our estimates of public sector expenditure for NCDs in Mongolia are based on Dugee et al [16] who used administrative data on utilization of public sector health services related to NCDs, and estimated public subsidies per unit of service. Utilization information included the number of discharges and length of stay per inpatient visit, and specialist outpatient visits, electronically recorded using ICD-10 classification, and GP visits from the Centre for Health Development of the Ministry of Health (MOH) and the Social Insurance General Office (SIGO). These administrative data also include information on inpatient admissions in private hospitals in Mongolia. Private OOP expenditure estimates for NCDs were based on data from the most recent WHO STEPs survey for Mongolia, supplemented by administrative records on public and private sectors’ hospital admissions and outpatient service use, and additional CVD-specific information from the Household Socioeconomic Survey (HSES) (Appendix SA2a and Appendix SA2b in **Online Supplementary Document(Online Supplementary Document)**).

The 2013 Mongolian STEPs survey is a nationally representative cross–sectional survey that sampled 6013 persons aged 15-64 years. The survey gathered household socioeconomic and demographic characteristics, NCD-related health service use and OOP spending on health services, drugs, accommodation and travel, and in-kind payments. Among STEPs surveys worldwide, the Mongolian survey is unique in having included questions on OOP health spending. The survey used the World Health Organization’s (WHO) standardized questionnaires and measurement protocols [32]. The WHO STEPs surveillance team oversaw survey implementation and provided direct technical support, and the survey data are generally considered to be high quality (Appendix SA1 in **Online Supplementary Document(Online Supplementary Document)**).

The administrative data we used for utilization of health care services are electronically recorded and shared between different levels of Ministry of Health facilities and ultimately the Centre for Health Development. Regular monthly checks ensure at the very least that the records are both consistent and complete in the system. All tertiary hospitals and health departments in Mongolian districts and provinces are supported by a Health Statistics and Information Technology unit that conducts routine checks for quality (Appendix SA1 in **Online Supplementary Document(Online Supplementary Document)**).

The HSES is a nationally representative survey of 16 200 households (56 791 individuals) conducted every year in Mongolia by the National Statistical Office. The main goal of the survey is to gather data on household expenditures and generally considered as generating good quality data. HSES data are used by many international agencies in their work. The World Health Organization’s National Health Accounts estimates for private OOP spending for Mongolia are largely based on the HSES survey data as is the World Bank’s “Health Equity and Financial Protection report for Mongolia” [33] (Appendix SA1 in **Online Supplementary Document(Online Supplementary Document)**).

### Estimates of out-of-pocket expenditures

We constructed sample-weighted estimates of OOP expenditures for NCDs using STEPs data. Private OOP expenditures on NCDs were estimated by region and type of service used and per utilization, separately for private and public sectors. Because, STEPs estimates of service use equalled 79.4% of total discharges and 90% percent of total outpatient visits in administrative data (73.6% of specialist visits and 97.0% of GP visits) among the age group 15-64, our survey-based utilization estimates were adjusted upwards using administrative records by the inverse of these rates for the age group 15-64.

OOP spending for specific NCDs was estimated as follows for the age group 15-64. The STEPS data set includes information on hospital discharges and associated OOP expenditures for CVDs and diabetes mellitus. This information was used to derive OOP spending per hospital discharge for CVD and diabetes mellitus. Because we could not separate out OOP payments for cancer discharges from OOP payments for COPD discharges in the STEPs survey, we assumed that OOP expenditure per discharge was the same in both cases. Estimates of discharges from administrative data sources by health condition, service provider and location, with corresponding information on OOP per discharge from the STEPs survey were then used to estimate OOP payments for hospital discharges by health condition, public and private sector, and by region (Appendix SA3a and Appendix SA3b in the **Online Supplementary Document(Online Supplementary Document)**).

Average OOP for a specialist outpatient visit for the age-group 15-64 was separately estimated for public and private facilities and region using the STEPs survey. Because STEPs data distinguished visits by public and private provider and by condition, OOP spending for a specialist outpatient visit was assumed to be the same for all NCDs, although different for public and private services.

OOP drug expenditures for outpatient services (GP visits were reported in the STEPs survey, along with associated outpatient OOP spending) were estimated for all regions separately for CVDs and diabetes mellitus, as this was explicitly distinguished in the survey. For cancer and COPD, per unit drug expenditures were assumed to be the same.

The STEPs survey is limited to individuals 15-64 years old. Thus aggregates of NCD spending based on STEPs data will underestimate OOP spending on NCDs because they exclude individuals 65 years and over, who accounted for nearly one-third of NCD-related hospital admissions and one-fourth of outpatient visits in 2013 in Mongolia, as per administrative records. Data from the Household Socioeconomic Survey (HSES) of 2014 shows that OOP expenditures for the 65+ age group exceeded 10% of total household OOP spending on health, indicating the significant share of OOP accounted for by this group.

To account for OOP spending on NCDs among people aged 65+, we estimated their inpatient admissions and specialist outpatient visits for each region and by health condition from administrative data. Relative shares of public and private sector service use were derived from HSES 2014, separately for inpatient care and specialist outpatient service use and for each region. Because public sector inpatients over 65 are exempt from fees, no OOP expenditures were assumed for their public-sector inpatient care use. For 65+ individuals using private inpatient care, OOP payment per discharge for a specific NCD was assumed to be equal to per discharge OOP payment for the age-group 15-64 in the STEPs survey. Similarly, OOP expenditures per specialist outpatient visit for people 65+ were assumed to be the same as for the 15-64 age-group in the STEPs survey.

The calculations above did not account for GP visits among people aged 65 years and over. Data from HSES 2014 showed that people aged 65+ accounted for 32.4% of all CVD-related GP visits. We assumed this proportion to hold for all NCDs and used this to derive the number of GP visits of 65+ individuals based on the GP utilization information for each NCD already available for the 15-64 age group. Of those aged 65+ undertaking GP visits, about 70% (based on the self-reported share of people incurring OOP spending in HSES, 2014) were assumed to incur OOP payments on drugs (GP consultation is free, but GPs do not dispense drugs, so any reported OOP was assumed to be on drugs). OOP expenditure on drugs per visit and by condition among people aged 65+ was assumed to be the same as reported in the STEPs survey for the age category 15-64.

Finally, for assessing the distribution of NCD spending by socioeconomic status, we constructed a measure of individuals’ standard of living (SLI) based on asset ownership and living condition [34] information on 17 variables for which data was collected in the STEPs survey, using principal component analysis (Appendix SA4a and Appendix SA4b in **Online Supplementary Document(Online Supplementary Document)**).

## RESULTS

In 2013, Mongolia spent an estimated 274.4 billion MNT (US$ 180.0 million) on health services for the 4 NCDs, accounting for 34.1% of total health expenditure (THE). About 40.0% (MNT 110.0 billion) of total NCD spending was on inpatient care, 45.6% on outpatient care and diagnostics (MNT 125.2 billion), 14.0% (MNT 38.0 billion) for drugs and the remainder (0.4%) for health promotion and surveillance.

**Figure 1:**
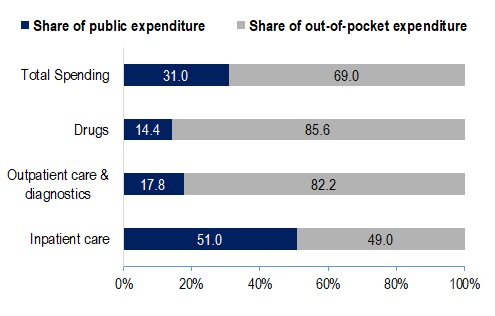
Expenditure share of public and private funding for total non-communicable disease (NCDs) spending, by type of service. Source: Authors’ analysis of data from the following sources: Public Expenditure Estimates are from Dugee et al, 2017 [16]; Out-of-Pocket Expenditures are based on WHO STEPs survey, 2013 for Mongolia and Administrative Dataset of Health Service Use from the Centre for Health Development of MOH of Mongolia.

**Figure 1** shows that public sources financed 51.0% of inpatient spending and OOP payments financed 82.2% of outpatient care and diagnostics and 85.6% of the drugs prescribed by ambulatory care services. Overall, OOP payments funded almost two-thirds of the estimated NCD spending in Mongolia.

**Figure 2:**
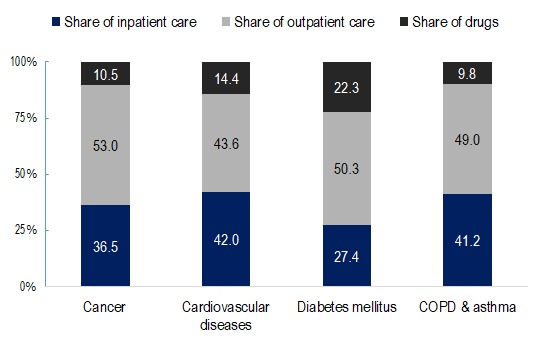
Percentage share of total expenditures of the 4 major NCDs by type of service. Source**:** Authors’ analysis of data from the following sources: Public Expenditure Estimates are from Dugee et al, 2017 [16]; Out-of-Pocket Expenditure estimates are based on WHO STEPs survey, 2013 for Mongolia and Administrative Data set of Health Service Use from the Centre for Health Development of MOH.

Expenditures for CVD were 24.2% of total health spending in 2013. Cancer-related health care spending was 5.1% of total expenditures, with COPD and Asthma, and Diabetes Mellitus accounting for about 2.6% and 2.1% of total health expenditures, respectively. **Figure 2** describes the share of total expenditures for each condition by type of service. Inpatient share in expenditures was highest for CVD (42.0%), whereas the largest fraction (51.6%) of cancer spending was on outpatient care. About one-third (27.9%) of expenditures for diabetes mellitus was on drugs.

**Table 1** presents information on public and private OOP spending per capita on services for NCDs in major Mongolian regions and the national capital (Ulan Bataar). Ulan Bataar had the highest per-capita public spending on NCDs, whereas the Northwestern region had the highest per capita OOP spending.

**Table 1:** Public and private spending per capita on major NCDs, by region in Mongolia (in US$)*

Region/condition	Public	Private	All NCDs
Western region	19.2	49.9	69.1
Khangai	17.0	43.7	60.7
Central	14.4	38.9	53.3
Eastern	16.1	44.0	60.2
**Province total**	**16.6**	**43.7**	**60.4**
Ulan Bataar†	21.2	40.8	62.0

NCDs – non-communicable diseases

*Source: Authors’ analysis of data from the following sources: Public Expenditure Estimates are from Dugee et al, 2017 [16]; Out-of-Pocket Expenditures are based on WHO STEPs survey, 2013 for Mongolia and Administrative Data set of Health Service Use from the Centre for Health Development of Ministry of Health.

†Ulan Bataar is the capital city where 47.0% of the Mongolian population reside. Provincial regions are in the order of a distance to the capital city from the farthest Western ( ~ 2000 km) to the nearest Eastern ( ~ 300km) region.

**Figure 3:**
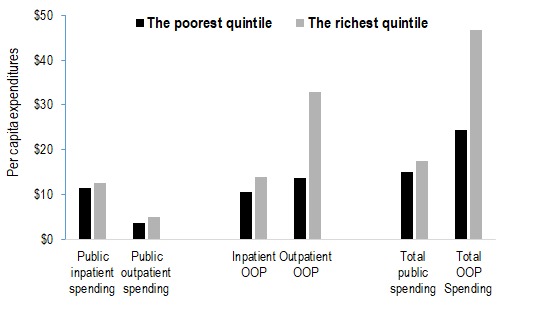
Distribution of public subsidy and private out of pocket expenditures on major NCDs in Mongolia, by level of care and socioeconomic status (per capita expenditure in US$). Source***:*** Authors’ analysis of data from the following sources: Public Expenditure Estimates are from Dugee et all, 2017 [16]; Out-of-Pocket Expenditures are based on WHO STEPs survey, 2013 for Mongolia and Administrative Data set of Health Service Use from the Centre for Health Development of MOH; Asset based quintiles constructed used data from the STEPs survey and the Household Socioeconomic survey, 2014 from the National Bureau of Statistics.

**Figure 3** shows annual public sector and OOP health spending per capita for NCDs by socioeconomic status. Public expenditures on services for major NCDs were nearly equally distributed across socioeconomic groups. However, public outpatient spending was slightly favourable to richest quintile, mainly due to the distribution of specialist outpatient spending, where its share was double that of the poorest quintile.

Private OOP expenditures on major NCDs were dominated by richer groups. The main driver was OOP payments for outpatient services, for which the share of the richest quintile was 2.4 times that of the poorest quintile. Overall, 11.5% of all OOP spending was accounted for by the poorest quintile and 22.1% was for health services used by the richest quintile.

**Figure 4:**
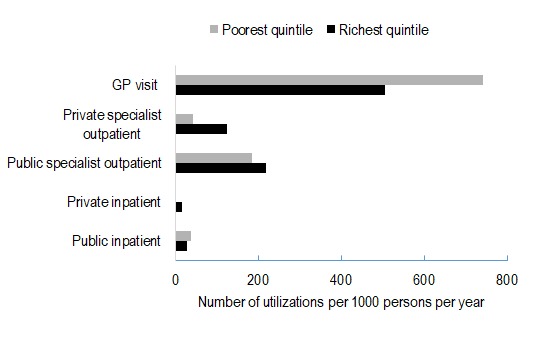
Use of inpatient and outpatient service use, by type of provider and socioeconomic status. Source: Authors’ analysis of data from the following sources: Public Expenditure Estimates are from Dugee et al, 2017 [16]; Out-of-Pocket Expenditures are based on WHO STEPs survey, 2013 for Mongolia and Administrative Dataset of Health Service Use from the Centre for Health Development of MOH of Mongolia.

**Figure 4** reports GP visits, specialist outpatient visits and inpatient admissions by provider and socioeconomic status. The use of public sector inpatient care (per 1000 persons) was lowest among the rich, and private inpatient service use (per 1000 persons) rose with socioeconomic status.

Public and private sector specialist outpatient visits (per 1000 persons) were lower for the poorest quintile, relative to the top quintile. On the other hand, per capita public sector GP visits were highest in the poorest quintile.

## DISCUSSION

Ours is the first comprehensive analysis of medical spending associated with NCDs for Mongolia, the way such spending is financed, and its distribution across regions and across population groups of different socioeconomic status. We find NCDs in Mongolia accounted for more than one-third of total health expenditures in 2013. Moreover, a significant fraction of this expenditure was borne by households as OOP spending. NCD-related spending and OOP spending shares also varies by socioeconomic status and region.

CVD-related health spending is the major driver of NCD-spending in Mongolia, accounting for about 24.2% of total health expenditure in 2013, comparable to high-income countries [35]. Moreover, estimated expenditures for NCDs track their relative disease burden in Mongolia: the Global Burden of Disease study estimated that CVDs accounted for 19.3% of DALYs lost in 2013 in Mongolia, followed by 11.2% for cancer, 1.5% for COPD and 1.1% for diabetes [36].

OOP spending on NCDs in Mongolia is driven mainly by outpatient care, including on diagnostics and drugs, suggesting that some households may be financially vulnerable to NCDs. This situation is not unique. Even in Thailand, a country generally thought to have achieved universal health coverage, households with a member who experienced chronic diseases have a greater likelihood of incurring catastrophic OOP spending on health [37]. In India, OOP spending on chronic diseases accounted for 47.3% of total household OOP health spending in 2004, comparable to 53.0% estimated in our study [13,38]. In Sri Lanka, cancer treatment is predominantly publicly financed, similar to Mongolia, whereas treatments for heart disease, diabetes and asthma were mainly privately financed. About half of the OOP expenditures for CVDs, diabetes and asthma were on outpatient care and drugs [15]. A recent study that used the WHO Study on Global Aging and Adult Health (SAGE) data reported that a large share of the OOP burden from NCDs in China and India was on outpatient visits, whereas hospital visits were a major source of OOP spending on NCDs in Russia, with medicines constituting the largest shares of all OOP spending [39]. This indicates a need for effective health financing mechanisms for addressing spending on outpatient care and medicines in middle-income countries with high NCD burden. The studies also highlight the costs associated with specialized care on NCD management in these countries, suggesting a need for reconfiguring NCD prevention and curative care services, possibly through an integrated primary care system [15,37,38].

Our finding of a significant share of OOP spending for NCDs in Mongolia is striking, as it is considered a country with extensive population health coverage through a combination of social insurance and subsidized public provision [25,40]. In 2013, almost 97.7% of the population was covered by social insurance in Mongolia, in addition to publicly funded GPs and subsidized government hospital services. And Mongolian health policy prioritizes good quality and equitable health services for those in need and financial risk protection against ill health [28,40]. However, a reliance on co-payments for public sector services, and growth in private sector and informal payments in Mongolia appears to have undercut these objectives. There is some evidence that in the interest of revenue raising, patients are subject to unnecessary tests, diagnostics and numerous user charges [24,27,40,41].

Given that OOP expenses are largely being incurred by the better off, it is possible to argue that social protection mechanisms in Mongolia limit the exposure of the poor to financial implications of NCDs. However, the same finding also suggests that the financing of public service delivery is benefiting the better off, as also suggested by Tsolmongerel et al [27]. Our study adds additional nuances to existing findings on the distribution of public resources for health. We find that public sector funds for inpatient care for the major NCDs are relatively equitably distributed by socioeconomic status, mainly because admissions in rural primary level hospitals are free, and because of exemptions for poor and retirees from co-payments in higher level hospitals [29]. On the other hand, public sector allocations for outpatient care disproportionately reach better-off groups, reflecting their greater use of such services. The latter finding may also reflect co-payment requirements for outpatient diagnostics in public hospitals [29]. Moreover, OOP expenditures for NCDs were higher for richer households, reflecting their greater use of private care and health services overall. Our findings on OOP are consistent with previous work for Mongolia [42] and other Asian countries [43,44] that found the better-off incurring greater OOP payments, although the focus was not specifically on NCDs.

We highlight important regional differences. Public subsidies per capita for NCDs were highest in Ulan Bataar, whereas OOP health spending per capita was highest in remote Northwestern Mongolia. This potentially indicates inadequate public sector services for populations with NCDs in remote areas of Mongolia. STEPs data indicate that OOP payments for inpatient drugs are common among admitted patients in public facilities lacking drug supplies, particularly in rural areas. Incidentally, rural-urban differentials in access to health services and variations in health service access across different regions existed even in the pre-reform (Soviet) era due to uneven resource allocation [45]. Regional differences may also occur owing to a lack of health literacy and inadequate primary care, with rural populations seeking care in later stages of chronic diseases, requiring urban-based specialised care in hospitals [46]. Mongolian administrative data indicate that in 2013, one-fourth of the inpatients treated in specialized tertiary hospitals in Ulan Bataar were transferred from rural regions [31].

The study has important limitations. Survey data on self-reported prevalence, service use and OOP payments are potentially subject to recall bias and small sample error [44]. We were, however, able to cross-validate the survey-based NCD-specific service utilization and adjust estimates by taking account of administrative data on health service utilization. We were also unable to separate out OOP payments for cancers and COPD in our data, and had to assume that OOP expenditures per use (outpatient visit or inpatient admission) were the same for both. We suspect any resulting bias may be small since, at least for inpatient care, cancer treatment is heavily state subsidized in Mongolia relative to other NCDs.

On the flip side, a major strength of this study is its reliance on data sources that are quite unique: good quality administrative data on health service use by health condition and related information on public spending, and a unique data set based on the only STEPs survey that gathered information on NCD-related health service use and OOP spending; and supplemental household surveys that made cross-validation of the STEPs estimates possible. There was reasonable agreement in the estimate of inpatient and outpatient service utilization for CVD and diabetes mellitus patients between the STEPs data and administrative data. Moreover, the STEPs survey based estimates of CVD related out-of-pocket expenditures on outpatient care corresponded well with the HSES estimate of OOP expenditures for cardiovascular outpatient caregiven the context of the downward biasedness of health expenditure estimates based on surveys of consumption expenditure.

## CONCLUSIONS

The findings of our study were based on triangulating multiple data sources that allow for consistency checks across a variety of sources. Yet the need to provide more robust information calls attention to the need for greater consistency across surveys in terms of disease and health spending subcategories. Our finding of a high share of OOP spending for NCDs in Mongolia which ostensibly enjoys universal health coverage, provides an important cautionary tale for LMICs in a similar situation. Improvement in the quality of services at the primary care level and rural health care facilities, where the poor mainly attend, is desirable together with an effective exemption policy for user fees at higher level hospitals. Investments in prevention programs under the Mongolian NCD strategy may need further strengthening. Heightened scrutiny of OOP payments in public facilities and strategic purchasing that allows for more coordination in care provided by public and private sectors may also help control OOP health expenditure growth.
