# Supplementary material for: Who is bearing the financial burden of non-communicable diseases in Mongolia?
Source: J Glob Health. 2018 Mar 15;8(1):010415. doi: 10.7189/jogh.08.010415 (PMC5857203; doi:10.7189/jogh.08.010415)
Supplement: Online Supplementary Document [file jogh-08-010415-s001.pdf]

Online Supplementary Document

Dugee et al. Who is bearing the financial burden of non-communicable diseases in Mongolia?

J Glob Health 2018;8:010415

Supplementary Appendix A1: DATA SOURCE AND QUALITY

There areD three main complementary sources of data used for generating the results reported in this paper:

A. The World Health Organization's STEPS survey administered by the Public Health Institute of the MOH

B. Routine administrative data from the Centre for Health Development (CHD) of the MOH

C. The Household Socio-Economic Survey (HSES) from the Mongolian Bureau of Statistics.

A. The STEPs survey (Stepwise Approach to Chronic Disease Surveillance)

STEPs survey is a repeated, cross-sectional survey, with the data for this paper coming from the third (and most recent) round of the survey conducted in 2013. The survey used stratified multistage cluster sampling to draw a nationally representative sample of adults aged 15-64 years. The Capital of Ulaanbaatar is an individual urban stratum and the remaining province/aimags are the rural strata. The final sample consists of one individual each (in the specified age-groups) from the randomly selected households. In 2013, the STEPs survey recruited 6,013 adults from all geographical areas of Mongolia. The survey used the World Health Organization's (WHO) standardized questionnaires and measurement protocols. The WHO STEPs surveillance team overlooked the survey implementation and provided direct technical support. STEPs surveys are the main data source of the WHO's Global Status Report on NCDs and "Global monitoring framework on NCDs" and generally considered to be high quality.

In our study, the STEPs survey was used to obtain estimates of OOP expenditures for NCDs and health service utilization estimates related to cardiovascular diseases and diabetes, by region and by type of provider. Estimates of OOP expenditures were based on respondent self-report and so may be subject to recall bias. And there is the possibility of sampling error. We were fortunate that apart from the STEPs survey that gathered OOP spending data on NCDs, there was a second contemporaneous survey (the Household Socioeconomic Survey or HSES) that gathered OOP data incurred by households for illness, although the information pertaining to NCDs was limited to cardiovascular disease (CVD) in HSES (see below). Given its limited nature, this information obviously could not be used to assess the consistency of all NCD spending reported in the STEPs data. However, we did find that the estimate of OOP expenditures on outpatient care for CVD patients in the STEPs survey corresponded reasonably with estimates derived from the independently conducted HSES survey that included a question on CVD OOP spending. For instance, per capita OOP expenditure per outpatient care for CVDs was estimated at 33,450 MNT (or $21.0) in the STEPs survey, whereas it was estimated at 30.488MNT (or $17.0) in the

HSES, 2014 for people of all ages and 27.438MNT (or $15.3) for people under 64 years. Here it is worthwhile noting that because of its focus on total expenditures (both health and non-health), surveys such as HSES are known to underestimate household spending on health, compared to surveys that report expenditures on health alone. While it is entirely possible to conduct sensitivity analyses for reported OOP spending based on the HSES (at least for CVD expenses), we do not recommend it, given these well-known biases.

Underpinning the estimates of OOP spending by type of NCD are healthcare utilization data on inpatient and outpatient services. These too, are based on respondent self-reports. These self-reports involve first inquiring about an individual's NCD status and then following up with questions on utilization of health services. As reported in our paper, STEPs survey estimates of service use amounted to about 80% of total discharges and 90% of total outpatient visits related to NCDs reported in administrative data people in the 15-64 age group. This serves as a good consistency check for STEPs data. Below we comment about the administrative data quality itself.

Separately, note that we also found good agreement in the estimates of inpatient and outpatient service utilization for CVD and diabetes patients between the STEPs data and administrative data. For instance, STEPs survey data yield estimated CVD and diabetes outpatient service visits to be 66.0% and 6.2% respectively of all NCD related outpatient visits. The corresponding proportions in the administrative records were 63.0% and 5.6%, respectively.

B. **Routine Administrative data from the CHD of the Ministry of Health.**

The data we used here are (a) hospitalization data (by ICD-10 classification); and (b) outpatient services used. There are a number of reasons that provide a basis for considering the health services utilization data to be of adequate quality. First, the data are electronically recorded and shared between different levels of the Ministry of Health and ultimately the CHD. Regular monthly checks ensure at the very least that the records are both consistent and (and for the records that exist) complete in the system. Currently all tertiary hospitals and health departments in Mongolian districts and provinces are supported by a Health Statistics and Information Technology unit that conducts routine checks for quality (mainly consistency and completeness).

The Mongolian government also has, under its Health Management Information System Development (HMISD) strategy (phase 1: 2005-2010 and phase 2: 2011-2015) and the E-health Strategy (2010-2014), focused on ensuring good quality information for decision making and integration of data across and between levels of the health system. These activities have included systematically addressing inconsistent data formats, coding schemes, and completeness in the data, and IT literacy and capacity. Periodic assessment on the timeliness, quality of information and feedback system is also conducted by the Department of Monitoring, Evaluation and Internal Auditing of the Ministry of Health.

C. **Household Socio-Economic survey (HSES) data from the Mongolian Bureau of Statistics.**

We used the Household Socio-Economic survey (HSES) data from the Mongolian Bureau of Statistics dataset for obtaining estimates of (a) health service utilization by type of provider for people 65+; (b) OOP expenditure for cardiovascular diseases

By most standards, HSES would be considered to generate good quality data. The HSES is a nationally representative, cross-sectional survey conducted every year in Mongolia by the National Statistical Office. The survey is conducted among randomly selected households and is representative at the national and provincial levels. In the most recent year for which data was gathered, 16200 households (and a total of 56791 individuals) were sampled. The main goal of the survey is to gather data on household expenditures on all goods and services. HSES data are used by many international agencies in their work, including the World Bank and the World Health Organization. For example, the World Health Organization’s National Health Accounts estimates for private OOP spending for Mongolia are largely based on the HSES survey data. The World Bank’ “Health Equity and Financial Protection report for Mongolia is also based on this survey. The survey also collects self-reported information on health service use by type of disease. However, among major NCDs, only cardiovascular diseases are included in the list of conditions for which expenditure and health service use data was collected. A drawback is that the utilization information by condition is asked of all outpatient service users, but only partially covers inpatient service use. It is for this reason (and also because surveys focused on consumption expenditures tend to underestimate the health component of such expenditures) that we relied mostly on STEPs data, and used HSES data mainly for consistency checks and to adjust estimates to account for age-groups not considered in STEPs – such as individuals 65 years and over.

Supplementary Appendix A2a. Health Service Utilizations related to NCDs for All Age, 2013 by Region, Type of Service and Sector

	Discharge		Specialist Outpatient visit		GP visit
	Public	Private	Public	Private	
Western	16,625	3,090	116,137	30,242	335,050
Khangai	20,716	3,854	168,823	43,863	366,244
Central	15,784	2,936	132,178	34,366	222,133
East	7,049	1,311	63,583	16,496	116,657
**Province total**	**60,174**	**11,191**	**480,721**	**124,966**	**1,040,084**
Ulan Bataar	58,007	18,725	309,816	143,211	855,731
**NCDs**	**118,181**	**29,916**	**790,537**	**268,177**	**1,895,815**
ALL DISEASE	612,058	121,452	6,221,554	2,136,313	8,218,528

Source: Authors calculation using administrative data from the Center for Health Development of MOH, WHO STEPs survey for Mongolia and the HSES from the National Statistical Office

Note: NCDs include cardiovascular diseases, cancer, diabetes mellitus, COPD and Asthma

Supplementary Appendix A2b. Health Service Utilizations related to NCDs for All Age, 2013 by Condition, Type of Service and Sector

	Discharge		Specialist Outpatient visit		GP visit
	Public	Private	Public	Private	
Cancer	16,453	767	120,690	48,155	274,563
Cardiovascular disease	87,380	25,967	566,654	183,620	1,2673,61
Diabetes Mellitus	4,224	1,022	39,803	14,652	205,564
COPD&Asthma	10,125	2,161	63,389	21,751	148,327
**NCDs**	**118,181**	**29,916**	**790,537**	**268,177**	**1,895,815**
ALL DISEASE	612,058	121,452	6,221,554	2,136,313	8,218,528

Source: Authors calculation using administrative data from the Center for Health Development of MOH, WHO STEPs survey for Mongolia and the HSES from the National Statistical Office

Supplementary Appendix A3a. Average OOP Spending per Discharge Estimated from the STEPs survey 2013, by Location and Condition

	Public hospital /in $US/					
	Western	Khangai	Central	East	Province average	Ulan Bataar
Cardiovascualr disease	145.2	212.4	151.1	154.1	165.4	281.7
Diabetes mellitus	373.3	234.2	117.4	241.6	244.4	162.3
Other diseases*	63.7	181.3	95.6	43.2	121.1	309.4
NCDs	154.7	205.6	140.9	117.4	162.1	283.1
	Private hospital /in $US/					
NCDs	450.3	376.8	367.5	312.5	420.1	832.1

Source: Authors calculation using WHO STEPs survey for Mongolia, administrative data from the Center for Health Development of MOH
*Other diseases include Cancer and COPD/Asthma; NCDs include Cardiovascular diseases, Cancer, Diabetes mellitus, COPD and Asthma

Note: Ulan Bataar is the capital city where 47.0% of the Mongolian population reside. Provincial regions are in the order of a distance to the capital city from the farthest Western (~2000 km) to the nearest Eastern (~300km) region.

Supplementary Appendix A3b. Average OOP Spending per Specialist Outpatient Visit and Drug Use, Estimated from the STEPs survey, by Location and Condition

	Average OOP expenditure, $US	
	Province average	Ulan Bataar
**Specialist Outpatient Visit**		
Public hospital	110.2	37.6
Private hospital	52.7	109.3
**Drug Use**		
Cardiovascular disease	10.1	5.4
Diabetes mellitus	21.0	6.3
Other diseases*	12.0	4.2
NCDs	11.3	5.2

Source: Authors calculation using WHO STEPs survey, administrative data from the Center for Health Development of MOH
*Other diseases include Cancer and COPD/Asthma; NCDs include Cardiovascular diseases, Cancer, Diabetes mellitus, COPD and Asthma

Note: In the provincial regions, public specialist outpatient visit is 4 times the private clinic visit (see Exhibit 2a).
The higher average cost per public outpatient visit in the province may reflect additional costs other than medical, mainly transportation cost of the travel from rural areas to the province centre or further to Regional Diagnostic & Treatment Centers or even further to the capital Ulan Bataar.

Supplementary Appendix A4a. Wealth index factor loading matrix and predicted scores among Mongolian

adults aged 15-64 years

Binary indicator variables	First component/Factor score	Predicted score
Electricity from grid	.438	.106
Electricity from generator	-.431	-.105
Television	.233	.057
Fixed line phone	.442	.108
Refrigerator	.541	.131
Computer	.614	.149
Running water	.706	.172
Flushing toilet	.701	.169
Radio	.133	.032
Mobile phone	.119	.029
Motorcycle	-.468	-.114
Bicycle	.075	.018
Five stock animals	-.372	-.090
Car	.380	.092
Savings	.337	.082
Land	.289	.070
Apartment	.706	.172
Ger	-.600	-.146
Sample size	6013	
% variance explained	20.5	
Eigenvalue	4.1	
Sampling adequacy	80.2	

Source: Authors calculation based on the WHO STEPs survey for Mongolia and Household Socioeconomic survey from the National Statisitical Office

*Track, house and other dwellings are excluded due to lack of sampling adequacy; Information on the house cannot be differentiated if its simple wooden house or luxury town house, so that its distributions across quintile did not have clear pattern.

Supplementary Appendix A4b. Proportional distributions of factor loadings across quintiles

Binary indicator variables/proportions binary =1	Quantile 1	Q2	Q3	Q4	Q5
Electricity from grid	77.8	97.8	98.1	99.3	99.9
Electricity from generator	29.5	5.9	1.9	2.1	0.5
Television	91.7	98.6	99.2	99.5	100
Fixed line phone	2.6	2.4	4.5	14.3	48.7
Refrigerator	56.8	96.8	99.0	99.6	100
Computer	8.5	35.1	68.3	86.6	95.9
Running water	0.5	0.6	3.1	27.3	96.7
Flushing toilet	0.4	0.6	2.9	28.1	95.0
Radio	10.8	9.4	13.3	16.9	25.6
Mobile phone	97.3	99.7	99.7	99.8	100
Motorcycle	57.7	32.4	17.9	6.8	2.1
Bicycle	17.4	24.6	26.6	28.2	26.5
Five stock animals	64.9	45.1	37.7	23.9	15.7
Car	21.1	36.7	53.9	69.8	75.7
Savings	31.5	43.5	58.6	71.2	79.5
Land	6.5	4.7	5.3	13.1	34.0
Apartment	0.0	0.5	2.3	26.7	95.9
Ger	81.1	47.8	26.2	6.2	0.0

Source: Authors calculation based on the WHO STEPs survey for Mongolia and Household Socioeconomic survey from the National Statisitical Office
